# A Longitudinal Study on Development and Validation of the Prostate Cancer Scale Among the System of Quality of Life Instruments for Patients with Cancer (QLICP-PR V2.0) Based on Classical Test Theory and Generalizability Theory

**DOI:** 10.1245/s10434-025-19044-4

**Published:** 2026-01-12

**Authors:** Wenxing Wei, Fengbo Guo, Tianci Zhang, Ying Chen, Gaofeng Li, Jiahong Luo, Chonghua Wan

**Affiliations:** 1 The First Dongguan Affiliated Hospital of Guangdong Medical University, Dongguan, China; 2 School of Humanities and Management, Research Center for Quality of Life and Applied Psychology, Guangdong Medical University, Dongguan, China; 3 School of Public Health, Kunming Medical University, Kunming, China; 4 The Third Affiliated Hospital of Kunming Medical University (Yunnan Tumor Hospital), Kunming, China

**Keywords:** Prostate cancer, Quality of life, Psychometric properties, Classical test theory, Generalizability theory

## Abstract

**Background:**

The purpose of this study was to develop and validate the prostate cancer scale among the system of quality-of-life instruments for patients with cancer (QLICP-PR V2.0) to provide a reliable tool for assessing quality of life changes and clinical outcomes.

**Patients and Methods:**

Application of programmed decision procedures, including multiple qualitative and quantitative analyses with general module and specific module combination pattern, were utilized to develop the QLICP-PR V2.0. On the basis of the data from 115 admitted and discharged patients with prostate cancer, the reliability, validity, and responsiveness of the QLICP-PR V2.0 scale were evaluated using correlation analysis, multi-trait scaling analysis, factor analysis, structural equation model, and paired *t*-tests, as well as combined with generalizability theory.

**Results:**

Cronbach’s alpha, test–retest reliability, and intra-class correlation coefficient of the total scale were 0.886, 0.896, and 0.889, respectively. Correlation analysis and factor analysis confirmed good construct validity and criterion-related validity. The score differences at all five domains and the total scale before and after the treatment were statistically significant (*P* < 0.05), with the SRM value ranging from − 1.152 to − 0.408, and the total scale was − 1.152. Generalizability theory results showed that the generalization coefficient of the five domains of the scale range from 0.575 to 0.764, and the reliability index between 0.529 and 0.748.

**Conclusions:**

The QLICP-PR V2.0 has good psychometric properties in reliability, validity, and responsiveness, and can be used to evaluate the quality of life of patients with prostate cancer.

Currently, there is a slowly increasing trend in prostate cancer incidence and mortality globally. There were approximately 1,276,000 prostate cancer cases and 359,000 prostate cancer deaths worldwide in 2018.^1^ There were about 1.4 million new cases and 375,000 deaths globally in 2020.^2,3^ In 2022, there were 1,466,718 new cases of prostate cancer and 396,773 deaths worldwide.^4,5^ Due to population growth and aging, the number of new cases of prostate cancer is projected to grow to nearly 2.3 million and 740,000 deaths by 2040.^6^ There are significant geographic differences in prostate cancer incidence globally. Higher rates are observed in regions such as North America, Australia, New Zealand, Europe, the Caribbean, and sub-Saharan Africa, while lower rates are found in areas such as Asia and North Africa.^7^ Meanwhile, mortality rates are also higher in the Caribbean and sub-Saharan Africa.^3^ Prostate cancer is one of the most common cancers in the world. The recognized risk factors are age, ethnicity, family history, and genetic history, and potentially modifiable risk factors are metabolic syndrome, obesity, and smoking.^8^ The prevalence of prostate cancer is closely related to age, with a higher incidence at older ages and the highest incidence in the 65–80 years age group.^9^ The 5-year survival rate of early prostate cancer is close to 100%, but the prognosis of advanced prostate cancer is poor. Therefore, we need to pay attention to the quality of life (QOL) of patients with prostate cancer.

The quality-of-life-specific scales developed abroad for prostate cancer mainly include the European Organization for Research and Treatment of Cancer Quality of Life Questionnaire-Prostate (EORTC QLQ-PR25),^10^ the Functional Assessment of Cancer Therapy-Prostate(FACT-P),^11,12^ the University of California Los Angeles-Prostate Cancer Index (UCLA-PCI),^13^ and the Expanded Prostate Cancer Index Composite (EPIC).^14^ The QLQ-PR25 questionnaire is typically used in conjunction with the general cancer assessment tool QLQ-C30. The QLQ-PR25 primarily assesses urinary, bowel, sexual symptoms and function, side effects of hormone therapy, and incontinence assistance,^10^ focusing on physical symptoms, emotional state, and sexual function.^15^ The FACT-P instrument consists of FACT-G and a 12-item prostate cancer subscale that assesses urinary function, bowel function, hormonal symptoms, and sexual function.^16^ In the EORTC QLQ-PR25 scale, all assessment items are presented as questions, whereas the FACT-P scale uses declarative sentences to express each item.^17^ The UCLA-PCI focuses on assessing sexual, urinary, and intestinal function and their troubles.^18^ The EPIC measures primarily the urinary, intestinal, sexual, and hormonal domains.^19^ EPIC was developed from UCLA-PCI and complements programs for urinary tract irritative and obstructive voiding symptoms and programs for hormonal symptoms.^14^

As cultural dependence of QOL, the foreign QOL scales for prostate cancer are diverse and have their own characteristics, but cultural differences make it difficult to fully reflect the situation of patients in China. For example, quality-of-life scales developed abroad pay more attention to religious beliefs and sexual life.^20^ In contrast, China pays more attention to family relationships, affection, food culture, and so on, which are not reflected in most western QOL instruments.^21^ Therefore, it is particularly important to develop a quality-of-life scale system that conforms to Chinese culture. Consequently, the quality of life instruments for cancer patients (QLICP) were systematically and independently developed on the basis of programmed decision-making procedures by drawing on the existing scales from abroad and focusing on Chinese characteristics.^21^ The system consists of a general module that measures a common part of the quality of life of patients with cancer, and some cancer-specific modules. The use of a combination of general and specific modules largely reduces the time and effort required to develop new scales.^22^ Compared with existing QOL instruments, the QLICP has a clearer structure and can better adapt to the characteristics of China culture. The updated version of QLICP (V2.0) includes the general module QLICP-GM (V2.0) and 22 cancer-specific scales such as nasopharyngeal cancer QLICP-NA (V2.0),^23^ breast cancer QLICP-BR (V2.0),^22^ cervical cancer QLICP-CE (V2.00),^24^ etc. Up to now, most scales of the QLICP (V2.0) have been developed and put into use.^25^

This study focuses on the development and validation of QLICP-PR V2.0 using classical test theory (CTT) and generalizability theory (GT). Classical test theory lays the theoretical foundation for scale development and validation, while generalizability theory further validates the reliability of scales by systematically controlling measurement error. Using the two theories, a more scientific and rational evaluation of the quality-of-life scale for prostate cancer will provide reference for the application and promotion of QLICP-PR V2.0.

## Patients and Methods

### Development of the QLICP-PR (V2.0)

QLICP-PR (V2.0) consists of the general module QLICP-GM (V2.0) and a prostate-cancer-specific module. QLICP-GM (V2.0) comprises 10 facets and 32 items, covering 4 domains: physical domain (2 facets, 8 items), psychological domain (3 facets, 9 items), social domain (3 facets, 8 items), and common symptoms and side effects (2 facets, 7 items). This general module has undergone systematic validation on the basis of classical test theory, item response theory, and generalizability theory, demonstrating good reliability, validity, and responsiveness.^26^

**Fig. 1:**
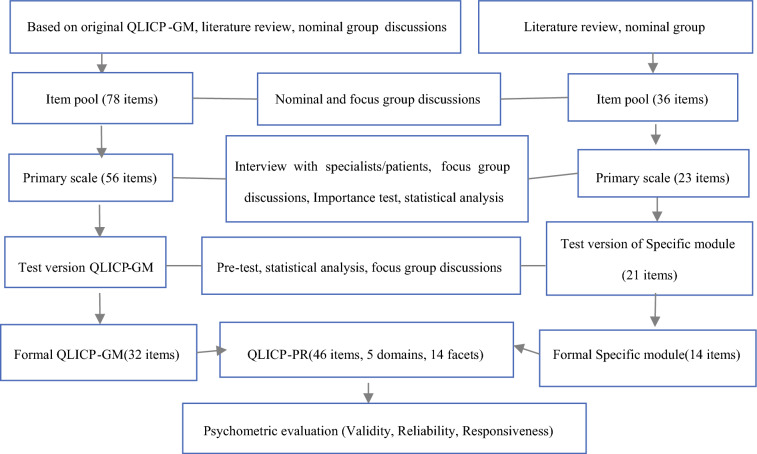
Steps toward development and validation procedure of QLICP-PR (V2.0)

The construction of specific modules followed steps similar to the development of the general module, employing a programmatic decision-making approach to screen items. The construction of specific modules utilized a programmatic decision-making approach similar to that of the general module to ensure the scientific rigor and clinical relevance of items. The specific development steps are as follows:

1. Establish a research team comprising nominal and focus groups of experts and scholars from oncology, urology, nursing, quality of life, psychology, and related fields.
2. Develop a conceptual framework for the prostate-cancer-specific module on the basis of quality-of-life principles and prostate cancer characteristics. This module is divided into urinary symptoms (URS), treatment-related symptoms (TRS), specific psychological effects (SPE), and sexual function problems (SFP).
3. Through systematic literature review, reference to relevant domestic and international scales, and expert panel discussions, a preliminary item pool of 36 items was proposed.
4. The research team conducted multiple rounds of discussions on the initial items, removing clinically irrelevant items and supplementing content deemed important by experts but not covered. This resulted in a preliminary specific module comprising 23 items.
5. Through interviews with patients with prostate cancer, healthcare providers, and relevant experts, as well as focus group discussions, the item wording and structure were further optimized, forming a test version of the specific module with 21 items.
6. Statistical analysis of pre-survey data and organized focus group discussions on item status led to retaining 14 items, forming the final version of the specific module.
7. The formal scale underwent systematic assessment of reliability, validity, and responsiveness using classical test theory and generalizability theory. The development process is shown in Fig. 1.

### Validation of the QLICP-PR (V2.0)

This is a longitudinal study involving three pre- and posttreatment measurements in patients hospitalized for prostate cancer, regardless of stage of disease and treatment.

### Participants

Yunnan Tumor Hospital was used as the investigation site to systematically screen patients with prostate cancer who met the criteria. Inclusion criteria included (1) patients who meet the diagnostic criteria for prostate cancer and have a clear diagnosis and (2) patients with elementary school education or above, good reading and comprehension skills, and who are able to fill out the questionnaire by themselves. Exclusion criteria included (1) patients with critical illness, combination of other serious diseases, serious mental diseases, etc.; (2) patients with cognitive dysfunction; (3) illiterate patients; and (4) patients who refused to participate in the study or were less cooperative.

### Survey Method

All investigators (doctors, nurses, medical graduates, and volunteers) received standardized training on the scale content and survey objectives. After obtaining written informed consent, patients completed the QLICP-PR (V2.0) by themselves. The first assessment was conducted on the day of admission; the second on days 2–3 to evaluate test–retest reliability; and the third prior to discharge to assess responsiveness.

### Scoring Rules

Each item is a five-level scoring system. Scoring options include not at all, a little bit, somewhat, quite a bit, and very much, which are counted as 1, 2, 3, 4, and 5 in that respective order. In the scale, items are divided into two types: positive items and negative items. Positive items do not need to be converted; the raw score is the item score. Negative items need to be transformed reversely, subtracting the item score from 6 to get the item raw score. The scores of items in the same domain are summed to obtain the domain score. The sum of the five domain scores constitutes the overall scale score. The standardized score (SS) was calculated by the formula SS = (raw score −  minimum) × 100/raw score, where the raw score (RS) is the maximum value minus the minimum value.

### Reliability Analysis

The reliability of the scale was analyzed using Cronbach’s alpha, test–retest reliability, intra-class correlation coefficient (ICC), and its 95% confidence interval.^27^ The internal consistency of the scale was mainly assessed by the Cronbach’s alpha coefficient for each domain. Pearson correlation coefficient and intra-class correlation coefficient between the first and second measurements were used to assess test–retest reliability of measures.^28^

### Validity Analysis

Validity was assessed through three aspects: content validity, construct validity, and criterion-related validity. Content validity was qualitatively evaluated by experts. The construct validity was analyzed by the correlation between items and domains and exploratory factor analysis (EFA), as well as confirmatory factor analysis (CFA) in structural equation modeling (SEM).^29^ Criterion-related validity was assessed by calculating the correlation coefficient between two scales. Multi-trait scale analysis is an important method used to test convergent and discriminant validity.^30^ There are usually two criteria: (1) convergent validity is considered to exist when the item-domain correlation is 0.40 or higher and (2) discriminant validity is indicated when the item-domain correlation is higher than the correlation with other domains.^31^

### Responsiveness Analysis

Sensitivity of the QLICP-PR V2.0 scale was assessed using paired *t*-tests and standardized response means (SRM);^32^ *t*-tests were used to analyze whether there was a significant difference in the mean change in scores before and after treatment. SRM was calculated as the ratio of the difference between the mean values before and after treatment to the standard deviation of the change values, reflecting the effect size. Standardized response mean is a valid measure of effect size; 0.20, 0.50, and 0.80 represent small, medium, and large reactivity, respectively.^33,34^

### Generalizability Theory Analysis

Generalizability theory (GT) was used to estimate the reliability and validity of measurements by analyzing the variance components of various potential sources of error in the measurement design.^23^ The research process is divided into two aspects: G-study and D-study, where G-study is generalizability study and D-study is decision research. The task of the G-study is to explore how to control and regulate the measurement errors by digging deeper into all the potential sources of measurement errors in the design and estimating the variance components of these errors.^22^ The main task of the D-study is to explore how to control and regulate the measurement errors by adjusting the various relationships in the measurement process on the basis of the error estimates from the G-study.^22^ When both the generalization coefficients (G coefficients) and reliability index (Φ coefficients) are greater than 0.6, the reliability of the scale is considered reliable.^32^

### Data Analysis Software

Reliability, validity, and responsiveness were analyzed using SPSS 26.0, structural equation modeling with Amos software, and GT with mGENOVA.

## Results

### Sociodemographic Characteristics of the Sample

This study investigated 115 men with prostate cancer. Among them, 94 (90.40%) were Han Chinese and 101 (87.80%) were married. Patients ranged in age from 49 to 86 years and the average age was 69.01 ± 7.69 years old. In terms of education, there were 24 cases (20.90%) with primary school education, 27 (23.50%) with junior middle school, 39 (33.90%) with senior high school, and 25 (21.70%) with university or above. The middle level of family economy accounted for 60.00%.

### Reliability

Table 1 presented the Cronbach’s alpha of the QLICD-PR (V2.0) scale as 0.886. Among the domains, the Cronbach’s alpha was close to 0.700 or above 0.700 for all domains, except for physical domain, at 0.575. Test–retest reliability (*r*) and intra-class correlation were above 0.8 for all domains except social domain.

**Table 1:** Reliability of the quality-of-life instrument QLICP-PR (V2.0) (*n* = 115 for *α*, *n* = 95 for r, ICC)

Domains/facets	Internal consistency coefficient *α*	Test–retest reliability correlation *r*	ICC (95% CI)
Physical domain (PHD)	0.575	0.853	0.848 (0.778–0.894)
Psychological domain (PSD)	0.764	0.880	0.871 (0.812–0.913)
Social domain (SOD)	0.680	0.595	0.591 (0.443–0.708)
Common symptoms and side effects (SSD)	0.749	0.910	0. 907 (0.863–0.937)
Sub-total (QLICP-GM)	0.867	0.873	0.867 (0.807–0.910)
Specific domain (SPD)	0.764	0.811	0.808 (0.725–0.868)
Total (TOT)	0.886	0.896	0.889 (0.838–0.925)

*ICC* intra-class correlation, *CI* confidence interval

### Content Validity

This study validates the content validity of the prostate cancer scale on the basis of literature review, maturity scale, and expert consultation. The scale includes five domains: physical domain, psychological domain, social domain, common symptoms and side effects, and specific domain. The items were set closely around the core content without important omissions, and the content integrity was good.

### Construct Validity

Correlation analysis, exploratory factor analysis, and confirmatory factor analysis (SEM) were used to test the construct validity. Correlation analysis was carried out by analyzing the correlations between the items and their own domains. Most items have higher correlation coefficients with their own domains than with other domains. However, some items (GPS2, GPS7, GSO4, etc.) showed weak correlation with their own domains (*r* < 0.40) (Table 2).

**Table 2:** Correlation coefficients r among items and domains of QLICP-PR (V2.0) (n = 115)

Items code	Items brief description	Physical function	Psychological function	Social function	Common symptoms	Specific module
GPH1	Appetite	0.447**	0.177	0.174	0.148	0.281**
GPH2	Sleep	0.527**	0.295**	0.200*	0.204*	0.356**
GPH3	Sexual function	0.419**	0.226*	0.122	0.164	0.280**
GPH4	Excrement	0.542**	0.137	0.252**	0.219*	0.277**
GPH5	Ability of daily living	0.635**	0.425**	0.459**	0.244**	0.255**
GPH6	Positive and optimistic	0.517**	0.378**	0.449**	0.218*	0.073
GPH7	Difficulty	0.620**	0.389**	0.210*	0.518**	0.399**
GPH8	Urinate	0.432**	0.064	0.236*	0.191*	0.393**
GPS1	Feel low or sad	0.253**	0.652**	0.217*	0.412**	0.231*
GPS2	Life interesting	0.219*	0.382**	0.353**	0.119	0.036
GPS3	Irritable	0.365**	0.663**	0.261**	0.385**	0.383**
GPS4	Memory deterioration	0.316**	0.531**	0.171	0.263**	0.223*
GPS5	Health deteriorated	0.411**	0.724**	0.177	0.529**	0.417**
GPS6	State of health	0.395**	0.678**	0.581**	0.216*	0.094
GPS7	Confidence	0.145	0.336**	0.423**	− 0.071	− 0.199*
GPS8	Disappointment	0.280**	0.721**	0.212*	0.516**	0.320**
GPS9	Attention	0.449**	0.642**	0.496**	0.244**	0.195*
GSO1	Family support	0.383**	0.403**	0.728**	0.173	0.049
GSO2	Other people’s care	0.255**	0.426**	0.664**	0.091	0.052
GSO3	Family role	0.413**	0.401**	0.735**	0.186*	0.061
GSO4	Finding help	− 0.085	− 0.080	0.280**	− 0.279**	− 0.244**
GSO5	Economic hardship	0.322**	0.290**	0.538**	0.318**	0.284**
GSO6	Labor status	0.260**	0.275**	0.398**	0.365**	0.308**
GSO7	Family/friend relationship	0.317**	0.338**	0.572**	0.160	− 0.005
GSO8	Social contact	0.412**	0.382**	0.689**	0.190*	0.132
GSS1	Nausea, vomiting	0.334**	0.284**	0.256**	0.621**	0.306**
GSS2	Lose hair	0.119	0.330**	0.125	0.482**	0.154
GSS3	Oral ulcer	0.199*	0.281**	0.163	0.610**	0.328**
GSS4	Pain	0.342**	0.291**	0.266**	0.598**	0.434**
GSS5	Thin	0.256**	0.349**	0.270**	0.712**	0.364**
GSS6	Dry mouth tastes bitter	0.371**	0.251**	0.100	0.747**	0.360**
GSS7	Fatigue	0.431**	0.450**	0.108	0.703**	0.415**
SPR1	Frequent urination	0.193*	− 0.039	− 0.034	0.270**	0.672**
SPR 2	Sleep	0.394**	0.238*	0.097	0.416**	0.780**
SPR 3	Urinary urgency symptoms	0.250**	0.167	0.018	0.360**	0.755**
SPR 4	Painful urination	0.334**	0.241**	0.144	0.344**	0.700**
SPR5	Blood in urine	0.260**	0.046	0.135	0.123	0.244**
SPR6	Difficulty urinating	0.202*	0.087	0.002	0.154	0.522**
SPR7	hot flashes	0.321**	0.188*	0.022	0.358**	0.436**
SPR8	Changes in secondary sexual characteristics	0.223*	0.126	0.064	0.303**	0.291**
SPR9	Low back pain	0.419**	0.229*	0.142	0.578**	0.491**
SPR10	Feeling a loss of manhood	0.260**	0.173	0.210*	0.207*	0.448**
SPR11	Worry about going out of town due to illness	0.449**	0.338**	0.221*	0.295**	0.651**
SPR12	Impact on sexual needs and interests	0.199*	0.185*	0.072	0.228*	0.314**
SPR13	Satisfaction with sexual life	0.161	0.134	0.113	− 0.072	**0.093**
SPR14	Problems with ejaculation	0.163	0.082	0.054	0.224*	0.412**

Correlations between each item and its designated scale are in bold type

**Significant at level of 0.01; *significant at level of 0.05

EFA results showed that the KMO value of the total scale was 0.733 and the *P*-value was less than 0.001. The KMO value of the general module was 0.762, and the variance contribution rate of 69.80%. The KMO value of the specific module was 0.771, with a variance contribution rate of 61.01%. Bartlett’s test for both general and specific modules was *P* < 0.001. Ten factors were extracted from the general module and four factors from the specific module; and the item loadings were all > 0.4, and the factor structure was consistent with the theoretical hypotheses.

CFA results by SEM showed that the modified general module model chi-squared/*df* was 1.743, GFI 0.925, CFI 0.938, TLI 0.896, IFI 0.941, and RMSEA 0.081. The measurement model of the specific module was fitted by confirmatory factor analysis of structural equation modeling. The results of the model fitting were chi-squared/*df* 1.614, GIF 0.879, CIF 0.897, TLI 0.868, IFI 0.901, and RMSEA 0.085.

### Criterion-Related Validity

Table 3 presented that the correlation coefficients between the QLICP-PR V2.0 and FACT-P (V4.0) scales are greater than 0.40 between the same or similar domains, and that each domain correlates significantly more with itself than the others.

**Table 3:** Correlation coefficients among domains scores of QLICP-PR (V2.0) and FACT-P (V4.0) (*n* = 115)

QLICP-PR	FACT-P					
	PWB	SWB	EWB	FWB	AC	PCS
Physical domain	0.620**	0.379**	0.375**	0.586**	0.678**	0.586**
Psychological domain	0.539**	0.357**	0.686**	0.522**	0.701**	0.365**
Social domain	0.493**	0.678**	0.379**	0.628**	0.767**	0.404**
Common symptoms and side effects	0.708**	0.084	0.489**	0.274**	0.510**	0.486**
Sub-total (QLICP-GM)	0.754**	0.478**	0.635**	0.643**	0.854**	0.581**
Specific domain	0.639**	0.044	0.370**	0.209*	0.415**	0.608**

*PWB* physical well-being, *SWB* social well-being, *EWB* emotional well-being, *FWB* functional well-being, *AC* additional concerns, *PCS* Prostate Cancer Subscale

^**^Significant at level of 0.01; *significant at level of 0.05

### Responsiveness

According to Table 4, at the domain level, statistically significant differences (*P* < 0.05) were observed across all domains. At the facet level, most facets of the scale also showed significant changes (*P* < 0.05), with the exception of sexual function problems. The standardized response mean (SRM) values ranged from − 1.152 to − 0.081 among different domains and facets. Specifically, the SRM was − 0.692 for the physical domain, 0.804 for the psychological domain, − 0.408 for the social domain, − 0.729 for common symptoms and side effects, − 0.958 for the general module, and − 1.062 for the specific module. The overall scale yielded an SRM of − 1.152. These results indicate that the score changes before and after treatment are clinically meaningful, supporting that the QLICP-PR (V2.0) exhibits moderate to good responsiveness.

**Table 4:** Responsiveness of the quality-of-life instrument QLICP-PR (V2.0) (*n* = 115)

QLICP-PR	Before treatment		After treatment		Differences		*t*	*P*	SRM
	Mean	SD	Mean	SD	Mean	SD			
**Physical domain**	**54.114**	**13.518**	**61.735**	**12.149**	− **7.621**	**11.010**	− **6.852**	**< 0.001**	**0.692**
Basic physiologic	48.571	14.466	57.959	13.059	− 9.388	13.048	− 7.123	< 0.001	0.719
functions									
Mobility and mobility	63.350	21.958	68.027	20.083	− 4.677	16.583	− 2.792	0.006	0.282
**Psychological domain**	**68.226**	**15.262**	**76.786**	**13.392**	− **8.560**	**10.641**	− **7.964**	**< 0.001**	**0.804**
Cognition	66.454	23.098	72.577	20.120	− 6.122	15.466	− 3.919	< 0.001	0.396
Emotion	70.000	18.500	79.082	15.604	− 9.082	13.742	− 6.542	< 0.001	0.661
Will and personality	65.561	21.318	75.255	19.660	− 9.694	20.349	− 4.716	< 0.001	0.476
**Social domain**	**66.932**	**14.246**	**71.460**	**12.689**	− **4.528**	**11.111**	− **4.034**	**< 0.001**	**0.408**
Interpersonal communication	72.577	19.219	76.148	15.553	− 3.571	16.548	− 2.137	0.035	0.216
Social support and security	63.967	15.357	68.048	14.187	− 4.082	13.495	− 2.994	0.003	0.302
Social role	67.219	20.817	73.597	18.643	− 6.378	16.178	− 3.902	< 0.001	0.394
**Common symptoms and side effects**	**77.843**	**17.124**	**89.249**	**12.184**	− **11.407**	**15.645**	− **7.218**	**< 0.001**	**0.729**
Common symptoms	68.282	23.223	82.908	16.204	− 14.626	23.663	− 6.119	< 0.001	0.618
Common side effects	85.013	16.879	94.005	11.595	− 8.992	13.789	− 6.456	< 0.001	0.652
**QLICP-GM**	**66.478**	**11.517**	**74.418**	**9.144**	− **7.940**	**8.285**	− **9.487**	**< 0.001**	**0.958**
**Specific domain**	**65.634**	**13.292**	**78.152**	**9.144**	− **12.518**	**11.788**	− **10.513**	**< 0.001**	**1.062**
Urinary symptoms	56.463	21.705	78.146	13.352	− 21.684	18.967	− 11.318	< 0.001	1.143
Treatment-related symptoms	82.398	18.915	91.412	9.070	− 9.014	17.614	− 5.066	< 0.001	0.512
Specific psychological effects	72.959	22.468	80.230	20.380	− 7.270	18.298	− 3.933	< 0.001	0.397
Sexual function problems	62.330	13.391	63.520	12.916	− 1.190	14.704	− 0.801	0.425	0.081
**Total (TOT)**	**66.221**	**10.772**	**75.555**	**8.153**	**−9.333**	**8.100**	− **11.407**	**< 0.001**	**1.152**

### G-Study Results

In the PHD, PSD, SOD, SSD, and SPD domains, the variance components of person-item interaction were 1.017, 0.900, 0.950, 0.878, and 1.155, respectively. The variance components of the five domains of the subjects ranged from 0.172 to 0.374, and those of the items ranged from 0.082 to 0.807, with the variability of the scale scores originating mainly from the person–item interaction. As presented in Table 5, the person–item interaction variance (P × I) is the main source of measurement error, indicating that the scale’s score differences are jointly affected by individual characteristics and item difficulty.

**Table 5:** Estimation of variance components in various domains in the *P* × *i*-designed G-study (*n* = 115)

	PHD	PSD	SOD	SSD	SPD
*P*	**0.172**	0.801	0.803	0.729	0.838
	0.188	**0.322**	0.733	0.656	0.424
	0.167	0.209	**0.252**	0.407	0.245
	0.185	0.227	0.125	**0.374**	0.725
	0.179	0.124	0.064	0.229	**0.267**
*I*	0.209				
		0.082			
			0.330		
				0.267	
					0.807
*P* *× i*	1.017				
		0.900			
			0.950		
				0.878	
					1.155

The elements on the main diagonal are the estimates of the variance components of each effect in the corresponding fields (shown in bold), the elements below the main diagonal are the estimates of the covariance components of the effects in different fields, and the elements above the main diagonal are the correlation coefficients between each field

*p* person, *i* item, *p × i* person-item, *PHD* physical domain, *PSD* psychological domain, *SOD* social domain, *SSD* common symptoms and side effects, *SPD* specific domain

### D-Study Results

The results in Table 6 present that the generalization coefficients of the five domains of the scale range from 0.575 to 0.764, and the reliability index ranges from 0.529 to 0.748. The generalization coefficients for the five domains of the scale range from 0.575 to 0.764, and the reliability indices range from 0.529 to 0.748. When the number of items in each domain reaches the target number, both G and Φ coefficients are raised to the desired level, which slows down with the increase of the number of items.

**Table 6:** *P* × *i*-designed D-study results of the various domains of QLICP-PR (V2.0)

Domain	Number of items	σ^2^ (*P*)	σ^2^(*I*)	σ^2^(PI)	σ^2^(*δ*)	σ^2^(Δ)	σ^2^(*X*_PI_)	Ε*ρ*^2^	*φ*
PHD	6	0.172	0.038	0.170	0.170	0.204	0.038	0.504	0.457
	7	0.172	0.033	0.145	0.145	0.175	0.033	0.542	0.495
	**8**	**0.172**	**0.029**	**0.127**	**0.127**	**0.153**	**0.029**	**0.575**	**0.529**
	9	0.172	0.026	0.113	0.113	0.136	0.026	0.603	0.558
PSD	7	0.322	0.016	0.128	0.128	0.140	0.016	0.715	0.697
	8	0.322	0.014	0.112	0.112	0.122	0.014	0.742	0.725
	**9**	**0.322**	**0.013**	**0.100**	**0.100**	**0.109**	**0.013**	**0.764**	**0.748**
	10	0.322	0.012	0.090	0.090	0.098	0.012	0.782	0.767
SOD	6	0.252	0.059	0.158	0.158	0.213	0.059	0.614	0.541
	7	0.252	0.051	0.136	0.136	0.183	0.051	0.650	0.579
	**8**	**0.252**	**0.044**	**0.119**	**0.119**	**0.160**	**0.044**	**0.680**	**0.611**
	9	0.252	0.040	0.106	0.106	0.142	0.040	0.705	0.639
SSD	6	0.373	0.049	0.146	0.146	0.191	0.049	0.719	0.662
	**7**	**0.374**	**0.042**	**0.125**	**0.125**	**0.164**	**0.042**	**0.749**	**0.696**
	8	0.374	0.038	0.110	0.110	0.143	0.038	0.773	0.723
	9	0.374	0.034	0.098	0.098	0.127	0.034	0.793	0.746
SPD	10	0.267	0.084	0.116	0.116	0.196	0.084	0.700	0.576
	12	0.267	0.070	0.096	0.096	0.164	0.070	0.734	0.620
	**14**	**0.267**	**0.061**	**0.083**	**0.083**	**0.140**	**0.061**	**0.764**	**0.656**
	15	0.267	0.057	0.077	0.077	0.130	0.057	0.776	0.671

*σ*^2^(*δ*) variance components of relative error, *σ*^2^(Δ) variance components of absolute error, *σ*^2^(XPI) variance components of error when estimating the universe score by using sample mean, *Eρ*^2^ generalizability coefficient, *Φ* index of dependability

Bold values represent results for the optimal number of items in the domain

## Discussion

This study aimed to develop and validate a culturally adapted quality-of-life scale, the QLICP-PR (V2.0), for Chinese patients with prostate cancer. The scale was developed through a programmed decision-making process, employing a modular design that combines general modules with prostate-cancer-specific modules. Psychometric evaluations of the QLICP-PR (V2.0) scale were conducted using classical test theory and generalizability theory. Results indicate that the QLICP-PR (V2.0) demonstrates good reliability, validity, and responsiveness among Chinese patients with prostate cancer, confirming its measurement properties as an effective assessment tool. Compared with existing international instruments, the QLICP-PR V2.0 scale offers several advantages.^25^ First, the general modules enable quality-of-life comparisons across different diseases. Second, specific modules precisely capture disease-specific issues, enhancing disease sensitivity and patient response rates. Third, the scale features a rational structure with a moderate number of items and a clear hierarchical organization (item→facet→domain→overall). Furthermore, QLICP-PR (V2.0) deeply integrates the Chinese cultural context, emphasizing characteristics such as family values, kinship bonds, and dietary culture, making it more culturally appropriate for Chinese patients. Therefore, the development and validation of the QLICP-PR (V2.0) scale holds significant importance.

Cronbach’s alpha and test–retest reliability methods were used to evaluate the reliability of the scale. Numerous domestic and international studies generally agree that Cronbach’s *α* ≥ 0.70 and test–retest reliability ≥ 0.80 meet acceptable standards.^30,35^ The results of this study indicate that both Cronbach’s alpha and test–retest reliability of the QLICP-PR(V2.0) scale approached 0.900, demonstrating high internal consistency and reliable measurement of patients’ quality of life. This confirms the scale’s robust stability. It must be acknowledged that certain domains exhibited suboptimal psychometric performance. The PHD demonstrated a coefficient *α* of 0.575, indicating poor internal consistency and stability. This stems from the domain’s composition of basic physiological functions and mobility/activity capabilities, whose combined nature results in “less consistent” performance within the scale.^36,37^ The lower Cronbach’s alpha (0.680) and test–retest reliability correlation (0.595) in the SOD align with Xu et al.’s findings.^38^ This may relate to factors such as conceptual ambiguity, cultural response bias (e.g., underreporting of social difficulties), or insufficient item relevance.

GT results further substantiate the reliability of the QLICP-PR (V2.0) scale. Overall, reliability indices are lower than G coefficients because they account for both interaction effects covered by generalization coefficients and primary error effects.^21^ In the QLICP-PR (V2.0) scale, most dimensions exhibit generalization coefficients and reliability indices above 0.60, indicating high measurement reliability across most dimensions and improved differentiation of quality-of-life levels among patients. The reliability of the social dimension was relatively low; increasing the number of items from 8 to 10 would achieve an acceptable reliability level. Generalizability test studies indicate that both the G-coefficient and the Φ-coefficient increase with the number of items.^23^

Regarding the validity of the QLICP-PR (V2.0) scale, we conducted validation across multiple dimensions. The development of QLICP-PR (V2.0) was grounded in the concept of quality of life, considering both the general characteristics of patients with cancer and the specific symptoms and psychosocial issues unique to patients with prostate cancer. Through systematic literature review and a multidisciplinary expert panel (encompassing clinical oncology, urology, psychology, and public health), items underwent multiple rounds of screening and revision using a procedural decision-making approach. This ensured the scale captures both universal quality-of-life dimensions and disease-specific challenges such as lower urinary tract symptoms and sexual dysfunction. Consequently, QLICP-PR (V2.0) demonstrates good content validity.

Regarding construct validity, correlation analyses revealed that the vast majority of items showed higher correlations within their own domains than with other domains, consistent with the fundamental assumptions of multi-trait scale analysis. This demonstrates good discriminant validity and convergent validity. However, some items exhibited weak correlations within their domains (*r* < 0.400) for the following reasons: The weak correlation of GPS2 likely stems from the broad concept of “life enjoyment,” which is susceptible to interference from multiple factors, including mood, cognition, and personality. Patient confidence in managing their disease fluctuates significantly with clinical factors such as pain and treatment stage, leading to reduced correlation between GPS7 and the PSD. The weak correlation of GSO4 with the social domain stems primarily from response biases influenced by the Chinese cultural context (e.g., avoiding burdening others, self-reliance). The low correlations of SPR5 and SPR8 with the prostate-cancer-specific module are mainly due to the low incidence of symptoms in the target population. Sexual issues are inherently private, and patients may withhold truthful responses due to shame, resulting in low correlations between SPR12 and SPR13 and the specific module.^23^ Exploratory factor analysis revealed that the factor structures of the general and specific modules largely align with theoretical expectations. Confirmatory factor analysis further confirmed that the model fit indices for both modules fall within acceptable ranges, indicating the scale possesses good construct validity.

Criterion-related validity analysis revealed statistically significant positive correlations between QLICP-PR (V2.0) and FACT-P (V4.0) across corresponding domains, indicating overall good criterion-related validity. Specifically, the highest correlation was observed between the total score of the QLICP-PR (V2.0) general module and the prostate cancer subscale score of FACT-P (*r* = 0.854), indicating high consistency in their overall assessment of prostate cancer patients’ quality of life. The specific modules of QLICP-PR (V2.0) also showed high correlations with the PCS dimension of FACT-P (*r* = 0.608). In summary, the QLICP-PR (V2.0) demonstrated good validity.

Regarding responsiveness, statistically significant differences (*P* < 0.05) were observed in all domains, subscales, and total scores before and after treatment, except for the sexual function problems facet. The SRM for the general module, specific module, and total scale were − 0.958, − 1.062, and − 1.152, respectively, indicating the scale’s strong responsiveness in effectively capturing treatment-induced changes in patients’ quality of life. No significant differences were observed in the sexual function subscale. This may be attributed to the relatively short observation period, the inherent stability of the symptoms themselves, and the highly private nature of the topic, which may have led to conservative responses from patients, thereby limiting the detection of changes.^21,23^

The study has several limitations. First, the relatively small sample size (*n* = 115) limits the generalizability of the findings. Second, recruitment was limited to a single tertiary hospital and exclusively included inpatients, resulting in insufficient representativeness and diversity of the study population. Future research should expand sample size, conduct multicenter collaborations, and incorporate more representative patient groups (e.g., outpatients, community-dwelling patients) to further validate the scale’s psychometric properties and applicability across different populations.

## Conclusions

The QLICP-PR (V2.0) is a quality-of-life assessment tool that meets psychometric requirements for reliability, validity, and responsiveness. Combining cultural specificity with methodological rigor, it serves as an effective instrument for evaluating the quality of life of Chinese patients with prostate cancer. This scale holds significant potential for clinical efficacy evaluation, public health policy development, and patient care.

## Data Availability

The datasets used and analyzed during the current study are available from the corresponding author on reasonable request.
